# The influence of smoke density on hearth location and activity areas at Lower Paleolithic Lazaret Cave, France

**DOI:** 10.1038/s41598-022-05517-z

**Published:** 2022-01-27

**Authors:** Yafit Kedar, Gil Kedar, Ran Barkai

**Affiliations:** 1 Department of Archaeology and Ancient Near Eastern Cultures, Tel-Aviv University, Tel-Aviv, Israel; 2 Independent Researcher, Tel-Aviv, Israel

**Keywords:** Archaeology, Cultural evolution, Software

## Abstract

We analyze the influence of hearth location and smoke dispersal on potential activity areas at Lower Paleolithic Lazaret Cave, France, focusing on archaeostratigraphic unit UA25, where a single hearth was unearthed, and GIS and activity area analysis were performed by the excavators. We simulated smoke dispersal from 16 hypothetical hearth locations and analyzed their effect on potential working spaces. Four activity zones were defined, according to the average smoke exposure recommendations from the World Health Organization (*WHO*) and Environmental Protection Agency (*EPA*). We found that the size of the low smoke density area and its distance from the hearth are the main parameters for choosing hearth location. The simulation results show an optimal hearth location zone of about 5 × 5m^2^, and it is precisely in this zone that the Lower Paleolithic humans of Lazaret Cave placed their hearth. We demonstrate that the optimal hearth location zone correlates not only with the archaeological hearth in UA25 but also with the locations of hearths in other layers. In addition, our smoke density analysis confirmed the detailed GIS and activity area reconstruction conducted by the excavators, strongly reinforcing their interpretation regarding the spatial organization of human behavior at Lazaret Cave.

## Introduction

In Paleolithic caves, the hearth was a focal point of group activity^1,2^, but its location had to be carefully chosen due to the harmful effects of smoke dispersal on the inhabitants. In this paper we apply our smoke dispersal model to a case study in order to verify whether early humans indeed considered smoke density as a parameter for space organization and activity partitioning in a cave. We take Paleolithic Lazaret Cave in France as a case study and analyze how smoke dispersal might have affected the placement of the hearth and related areas of human activity by applying computer-based simulations. Research on the habitual use of fire in the Paleolithic focuses mostly on understanding the advantages provided by the hearth, such as heat, protection, light, and a place for cooking, tool making, tool hafting, and social conversation^3–29^. The lively debate regarding fire management and early human ignition abilities should be noted in this context. Some suggest that hearths were active for a long duration without repeated ignition or even question the ability of early humans to produce fire at will, while others suggest that hearth ignition was controlled and recurrently practiced (for claims for and against, see, e.g.,^30–34^). Proponents of both views agree, however, that Paleolithic hearths were probably active for a duration of at least several hours^35,35–39^. In recognition of the importance of hearths at Paleolithic sites, several methods were developed to identify and locate ancient Paleolithic hearths. Some are based on chemical analysis of sediment while others use lithic distribution and other means^17,40–49^.

Chemical methods to locate the hearth can be direct or indirect^40,50^. Direct methods use microarchaeology analyses that identify the products of fire such as ash and calcite, which are dispersed in the surrounding space of the site^38,49,51,52^. Indirect methods analyze the effects of heat convection, which can be found up to several centimeters from the hearth itself. The heat from the hearth changes the chemical properties of the artifacts in its immediate vicinity, such as bones and flint items, as well as the properties of the sediments in contact with the hearth^40,42,53^.

Hearths can also be located by mapping the lithic distribution according to the "toss and drop" model^54^, or using the ring and sector method, which enhanced the "toss and drop" model by also considering the distribution of artifacts according to lithic type, weight, and prevailing wind direction^55,56^. Sorensen & Scherjon^57^ augmented the lithic distribution model with additional parameters, such as surface area, hearth size, and sedimentation rate, which greatly influence the visibility of archaeological fire signals.

In an experiment by Gentles & Smithson^58^, it was shown that a hearth lit for 20 min in the interior of a cave during the summer produced so much smoke as to prevent human occupation altogether. In a newer experiment^59^, the hearth produced smoke that quickly polluted the cave: after 30 min human presence was no longer possible and the fire had to be extinguished. The authors concluded that the hearth was not well placed. High density smoke causes burning eyes and respiratory problems, such as coughing^60,61^. A model that correlates between the cave structure, hearth location, season of use, and smoke height showed that hearth location has a major effect on the smoke height in a cave and is thus a major influence on potential human activity in areas affected by the smoke. According to this model, an optimal ventilation rate is obtained if the hearth is located between the center to the back wall of the cave^62,63^.

We chose one of the best known and studied Lower Paleolithic cave sites in Europe, Lazaret Cave, France. Lazaret Cave was meticulously excavated and analyzed^64–72^ and is not collapsed. We focused on layer UA25, since detailed research on artifact distribution and activity areas is available^73^. We simulated smoke density, using software-based smoke dispersal simulator, according to hearth location and cave structure, using an artificial grid system of 0.5 × 0.5 m squares to cover the entire cave area, and correlated smoke density with the activity areas suggested by the excavators on the basis of the archaeological remains. Smoke densities in different areas of the cave were designated according to the definition of the World Health Organization (*WHO*)^74^ and the Environmental Protection Agency (*EPA*)^75^. Following that, we defined potential long-duration occupation versus short-duration occupation areas according to the smoke density, while areas with the densest smoke are most likely unsuitable for human presence. We found good correlation between the smoke density and the proposed activity areas at level UA25. In addition, in order to understand which parameters influence the choice of hearth location in this layer, we simulated 16 different potential hearth locations distributed throughout the entire cave. We investigated the effect of each hypothetical hearth location on the size of the expected long-duration occupation area. We also calculated the extent of the warmth zone of each hearth, designated as the area less than 5 m distance from the hearth^58^. As expected, choosing the most appropriate location for a hearth is a complex decision, affecting the size of the area available for long-duration occupation and the extent of the beneficial warmth zone. Simulations show that the archaeological hearth in level UA25 is optimally located, since it creates a large, long-duration occupation area close to the hearth and a large area suitable for meat smoking, as previously suggested by the excavators. In the following, we also examine hearth locations in other layers from Lazaret Cave and analyze the optimality of their location.

## Methods

The hearth location analysis considers two main parameters: heat and smoke. The heat from an active hearth increases the surrounding temperature in the cave, allowing the area to be used for warmth, while the smoke density influences the potential for human occupation. In this section, we describe the air circulation model that we used for smoke density analysis as well as the temperature model used in our software-based smoke dispersal simulation study.

### Air circulation model

**Figure 1:**
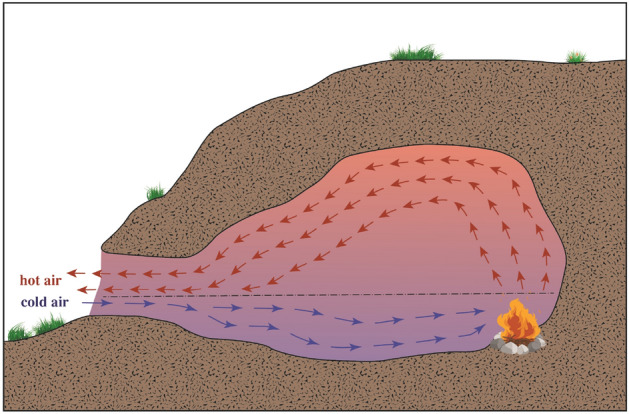
Smoke dispersal in a cave. Smoke is emitted towards the ceiling in the direction of the cave opening. The arrows represent air circulation, and the broken line represents the balance point between the cold and hot air flows (taken from^62^).

Smoke dispersal and smoke density in caves are calculated based on hearth location and cave dimensions. The cave mouth is a natural ventilation opening, and thus warm air is ventilated out of the cave through an upper layer, above the broken line in Fig. 1, while cold air enters the cave from a lower layer below the broken line^62^.

The air circulates due to the burning process, which increases the air temperature from the lower cave layer and passes it to the upper layer, resulting in increased pressure in the upper layer. The layer pressure is defined as^76^:

1 $$P=\frac{{m}_{i}RT}{V}$$

where P is the gas pressure in pascal units (N/m^2^), m_i_ is the layer mass, V is the layer volume, R is the gas constant, and T is the smoke temperature in Kelvin.

The air ventilation rate depends on the dimensions, average height (t), and average width (w) of the cave mouth. The cave ventilation rate can be defined as the air mass flow per second (*amps*), which is based on Bernoulli's equation:

2 $$amps=\underset{0}{\overset{t}{\int }}wC\sqrt{2\rho \Delta P\left(z\right)} dz$$

where *C* is the orifice coefficient taken to be 0.7^77^, ρ is the gas density, and ∆*P(z)* is the difference in air pressure (Eq. 1) at height *z*.

For a detailed mathematical model, the reader is directed to "Fire Dynamics Simulator Technical Reference Guide Volume 1: Mathematical Model" developed by NIST, USA^78^.

### Temperature

Heat, resulting from fuel combustion, is essential for warmth, cooking, and tool management^15,79–83^. Thermal energy from fuel combustion is transferred to the surroundings via convection or radiation^84^.

Gentles & Smithson^58^ measured the temperature in a cave at Creswell Crags, Nottinghamshire, England to analyze the effects of fire on the thermal micro-climate of the cave. They experimented with two hearth locations, one near the cave back wall and one at the cave entrance, and measured temperatures every 3 m during winter and summer. When the hearth was located at the back of the cave, the surrounding temperature linearly decreased to normal after a distance of 6 m from the hearth (both in summer and winter). When the hearth was located at the cave entrance, the cave temperature was barely affected both in winter and summer. In another experiment, at an open-air site, Hoare^84^ evaluated the heat convection and radiation correlated with hearth features, such as various fuel types. The measurements were conducted at air temperature of 11–13 °C and were taken every 10 min at distances of 1, 2 and 3 m from the fire at a height of 1 m above the hearth. For most of the examined fuel types, the measured temperature was similar, but burning times differed. For a 100 cm diameter hearth, peak temperatures at a 1 m distance ranged from 21.6 to 52 °C, average temperatures at a 2 m distance were 14 to 19.8 °C, and at 3 m the temperatures exceeded the ambient temperature by up to 2 °C. Beyond 3 m, there is no heating effect at an open-air site.

#### Site description—Lazaret Cave, Nice, France

Late Acheulian-Early Mousterian Lazaret Cave is located on the western slope of Mount Boron on the eastern border of the city of Nice, France and is dated to 230–37 kya^64,66–73^. The excavated sediments are associated with the last cold episode of the Middle Pleistocene, reflected by the large mammals and rodents and subsequent paleoecological data^73,85–87^. The multidisciplinary analyses conducted at the site revealed successive occupations by groups of hunters (of large herbivores, mainly red deer and ibex), who set up temporary camps and sometimes occupied the cave for more prolonged periods^70,85,86^. The stratigraphic sequence includes a sequence of Lower Paleolithic Acheulean deposits as well as the Acheulean/Mousterian transition during the course of MIS 6^71,88^. Five main archaeological stratigraphic units (A, B, C, D, E) were excavated, and further subdivided to 28 archaeological levels. The CII unit contains an Acheulean lithic assemblage with numerous handaxes and some rare Levallois debitage. Above this deposit, the CIII unit is attributed to the Mousterian. More than twenty pre-Neanderthal human remains have been discovered in unit C. One of the most important remains is a sub-adult frontal, discovered in August 2011 in UA28 and which presents a transitional morphology between *Homo heidelbergensis* and *Homo Neandertalensis*^73,85,89^.

The cave is 40 m long and 4 to 15 m wide, with a ceiling height of up to 15 m and an entrance width of about 3 m. The cave surface is about 290 m^2^. The excavation area was spread over 100 m^2^ close to the entrance of the cave and included excavation squares 1–17. This area was excavated to a depth of up to five meters. Excavation squares 18–26 were excavated only to the surface level, and squares 27–37 were never excavated^68,71,73^. In this paper, we focus on main unit C, since it contains several layers with hearths, with a specific focus on archaeostratigraphic unit 25 (UA25). Main stratigraphic unit C is divided to three sub-units: sub-unit CI, dated to 190–170 kya, designated as late Acheulean, sub-unit CII, dated to 170–150 kya, designated as late Acheulean, and sub-unit CIII, dated to 150–120 kya, designated as Mousterian of Acheulean tradition^64,71,85^. Stratigraphic sub-unit CII contains UA13 to UA28, and stratigraphic sub-unit CIII contains UA1 to UA12^85–87^.

**Figure 2:**
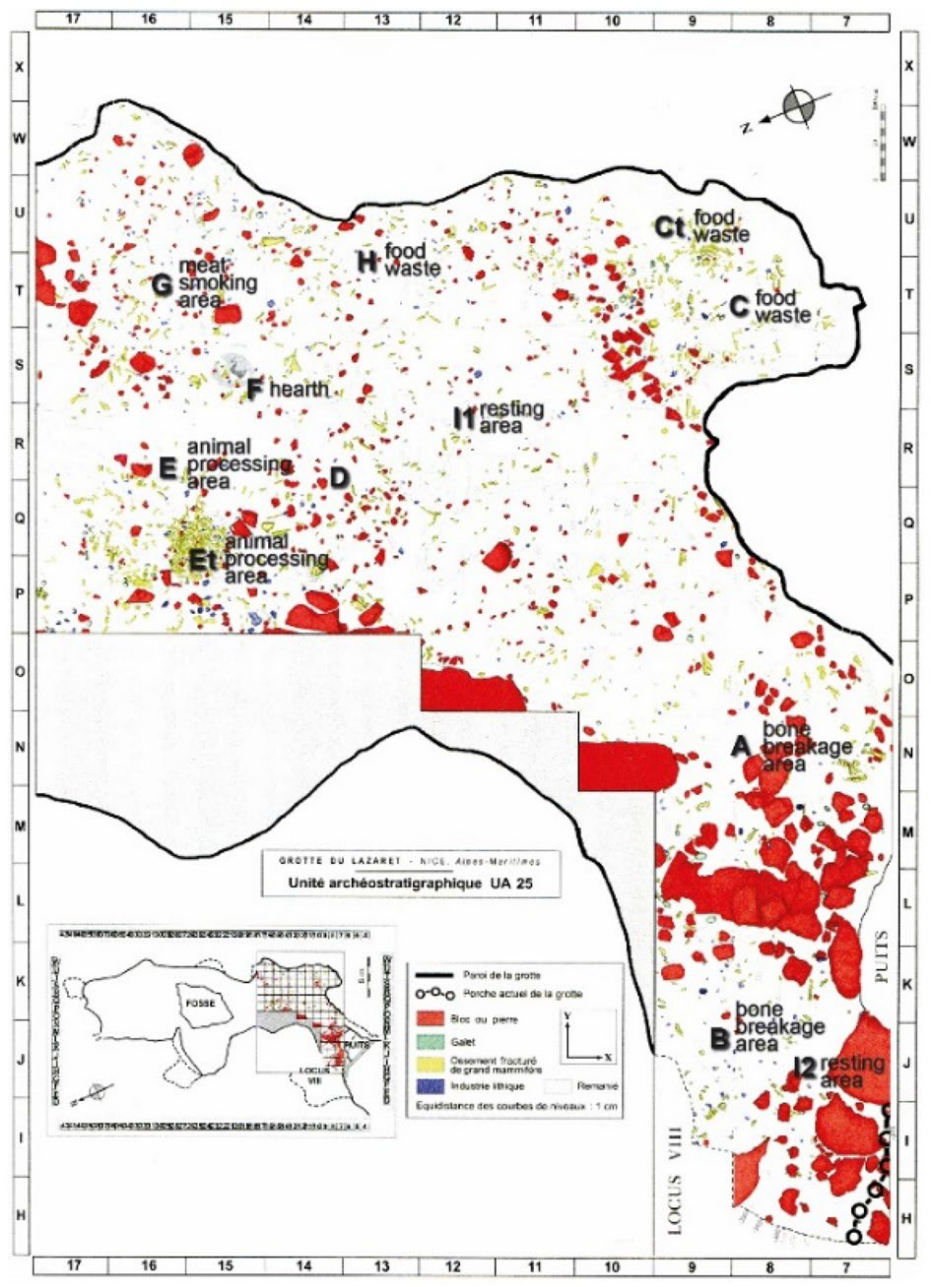
Lazaret cave, GIS map of UA25 with activity area, based on (^65^, p. 172). The activity areas were designated by the excavators on the basis of the archaeological evidence (red—block and stone green—pebble, yellow—large mammal bone, blue—lithic pieces) (courtesy of Prof. Henry de Lumley).

We chose level UA25 since it demonstrates unique preservation, exhibits a single hearth, and its meticulous study provided a detailed reconstruction of related activity areas. The excavated area is spread over ca. 86 m^2^ and the archaeological remains are mostly in situ^64^. A single hearth was unearthed in the cave's interior in Area F, which is located about 13 m from the entrance, as shown in Fig. 2^72^. The exceptional state of preservation of UA25 allowed the researchers to identify different processing stages of large game for culinary or other purposes and also several other distinct areas of human activity^70,86,87^.

The lithic and fauna assemblages are clearly the result of hunting-oriented activity^71,73^. UA25 contains 3260 large mammal remains, of which 1286 have been identified to the species level^71^. Evidence of activity was used to divide the level into different designated areas, as shown in Fig. 2. Areas A and B were interpreted as bone breakage areas^72^. In addition, many bifaces were found towards the cave entrance in area A, and Area B includes numerous lithic items, including large flakes and three handaxes. Along the east wall of the cave, areas C, Ct, and H are characterized by accumulated food waste. Area G, located behind the hearth, is thought to have been used for meat smoking and drying. Area E is comprised mostly of handaxes and pebbles, interpreted as tools used for animal processing: butchery and bone breaking. The Et heap contains two types of large sized bone waste: untreated bones and culinary remains. It is thus interpreted as a waste deposit area. In Area F, ashes located in excavation square S15 indicate a small-size hearth. Ash analysis revealed the burning of terrestrial and marine plants. The excavators proposed that the hearth was used to maintain a smoke fire at low temperature. Areas I1 and I2 contain lower artifact densities with a pronounced presence of tiny seashells, and the authors suggest that this was the resting area. In area I1 beddings comprised of marine and terrestrial plants were found^64^.

The researchers suggested that the hearth produced low-temperature smoke intended mostly for meat preservation^67,72^. They concluded that hunting and meat preservation usually took place in autumn and winter, between October and December, in order to stock up on food before the cold sets in^72^.

Hearths were also identified in other archaeostratigraphic units of Lazaret cave. In UA26, four small hearths were found: in squares P10 (20 cm diameter), squares R12/R13 (45 cm diameter), square S15 (20 cm diameter), and square R16 (45 cm diameter). In UA27 hearths were located in square Q11 (15 cm diameter), square P13 (15 cm diameter), square Q13 (70 cm diameter), square R14 (25 cm diameter), square T15 (20 cm diameter), and square T17. In UA28, a single hearth was located in square O10 (35 cm diameter). In UA29 hearths with a diameter of 30 cm were located in squares P9, Q10, Q12 & S16^65^.

#### Simulation description

In order to evaluate smoke density in Lazaret Cave, we used the Fire Dynamic Simulator (*FDS*) application. The National Institute of Standards and Technologies (NIST) developed FDS for simulating fire and temperature reactions in closed spaces^78,90^. FDS is based on a computational fluid dynamics model of fire-driven fluid flow. It solves thermally driven flow equations with an emphasis on smoke and heat transport from fires, and it was used by us^62,63^ to analyze the relationship between smoke height, cave dimensions, and hearth location in Paleolithic caves and rockshelters. In addition, FDS was used to simulate fire events at Chauvet-Pont d'Arc Cave, France, and its results were compared to an experiment conducted in an underground limestone quarry^91^.

Hearth characteristics such as heat release rate (*HRR*) and smoke release rate were defined. The amount of smoke dispersed from a hearth depends on the hearth size and hearth heat release rate. The HRR value is correlated with the amount of used wood (and/or other burning materials). We defined the HRR value to be 90 kW, as in^91^, who performed a fire experiment in a limestone quarry similar in size to the Megaceros Gallery of Chauvet-Pont d'Arc, France, using dry Scots pine (*Pinus sylvestris*)^92^. For the smoke release rate, we used the suggested parameters for dry wood (amount of smoke = 0.015%)^93^.

**Figure 3:**
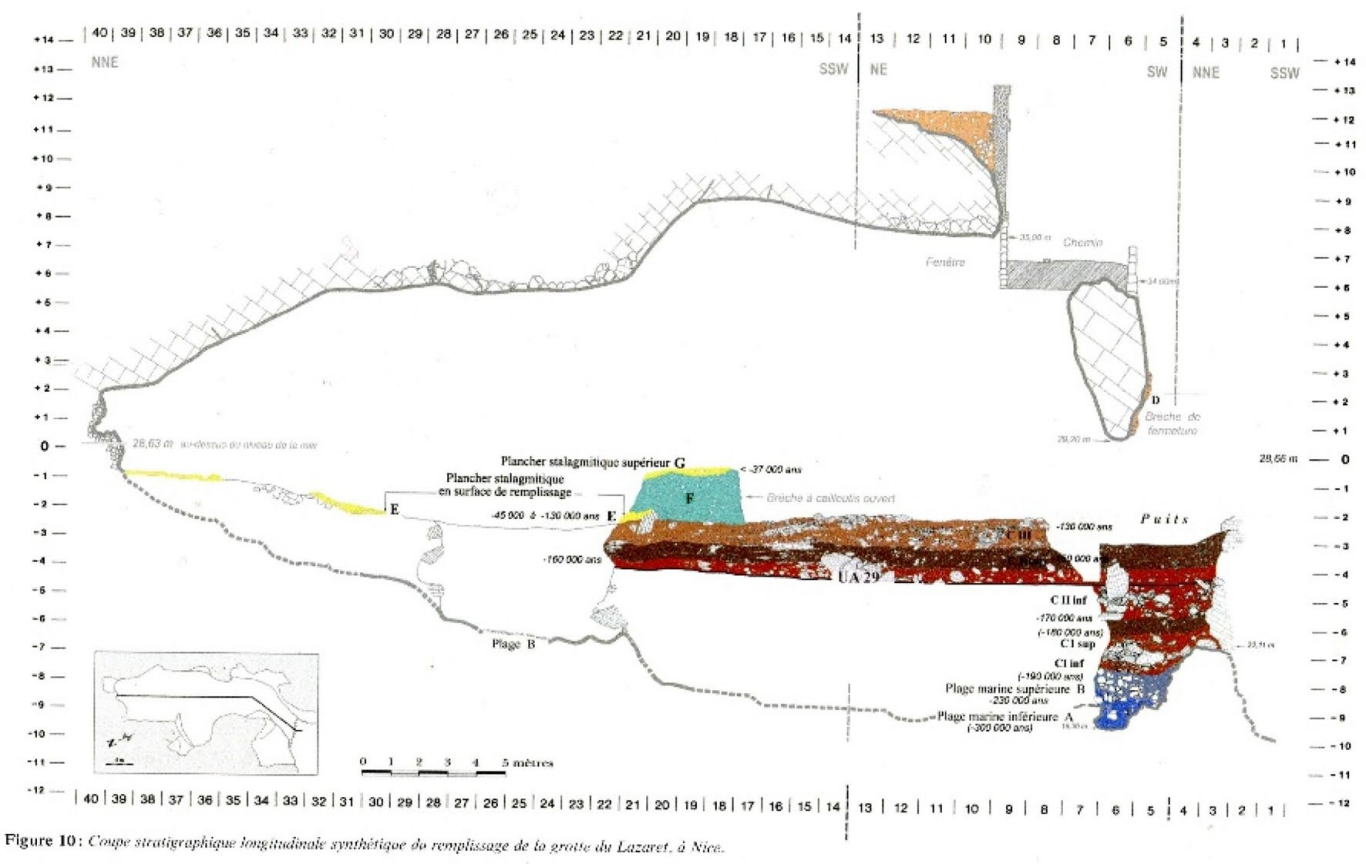
Lazaret Cave section diagram^85^. (Courtesy of Prof. Henry de Lumley).

**Figure 4:**
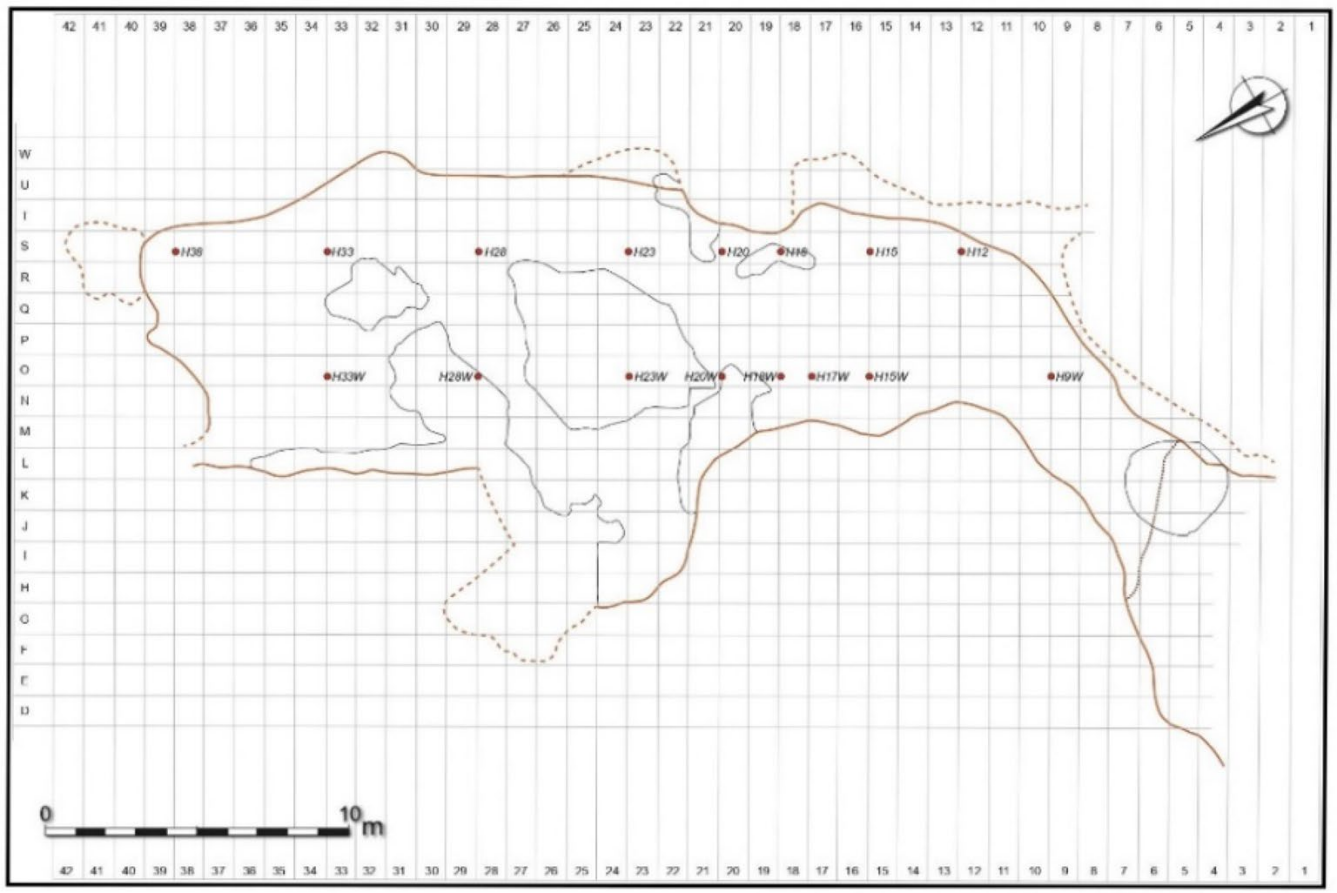
Lazaret Cave map with the simulated hypothetical hearth locations, based on (^69^, p. 40). (Courtesy of Prof. Henry de Lumley).

In order to analyze the potential influence of various hearth locations on smoke density in Lazaret Cave, we simulated the published dimensions of the cave^66,69^, as shown in Figs. 3 and 4. We placed hypothetical hearths at 16 different locations, termed Hxx (Hearth at horizontal square xx), where the HxxW hearths are located on the cave's west side and the Hxx hearths are located on the east side. H15 is the location of the actual hearth at archaeostratigraphic unit UA 25. The simulated hearths were located from the cave back wall up to the cave entrance in steps of about 5 m. In the middle zone of the cave we simulated the hearths at the two sides of the space, as presented in Fig. 4. We simulated an hour-long use of a hearth, since, according to our simulations, smoke density will have stabilized by this time. We configured about 6000 virtual smoke density sensors all over the cave, from the entrance to the cave back wall, at heights of 1.5 m (standing height), 1 m (sitting height), and 0.5 m (sleeping height), with 0.5 m between the sensors. Each sensor recorded the smoke density in kg/m^3^.

The simulation results were analyzed using Microsoft Excel and can be visualized using the Smokeview application^78^.

#### Simulation results

The cave was divided to four sub-areas, following smoke exposure guidelines published by the World Health Organization (*WHO*)^74^ and the Environmental Protection Agency (*EPA*)^75^. These are modern health guidelines for Western populations. While we acknowledge that Paleolithic human tolerance for smoke may have been different, we currently have no measure for estimating such differences. The WHO and EPA guidelines recommend a maximum smoke density exposure of around 10 µg/m^3^ for 24 h, which translates to a smoke density of around 10e-9 kg/m^3^ in the simulation units. The four smoke density zones were defined as follows:

Red area: smoke density higher than 0.000001 (1e-6), termed non-occupational area. In this area the smoke density is at least 1000 times higher than the above guidelines.

Yellow area: smoke density between 1e−6 and 1e−9, termed suitable for short-duration occupation of several minutes^58,59,94^. In this area the smoke density is up to 1000 times higher than the guidelines.

Green area: smoke density between 1e−9 and 1e−12, within WHO/EPA limits, termed suitable for longer-duration occupation of several hours/days. In this area the smoke density is up to 1000 times lower than the guidelines.

Blue area: smoke density lower than 1e−12, termed suitable for long-duration occupation. In this area the smoke density is more than 1000 times lower than the guidelines.

**Figure 5:**
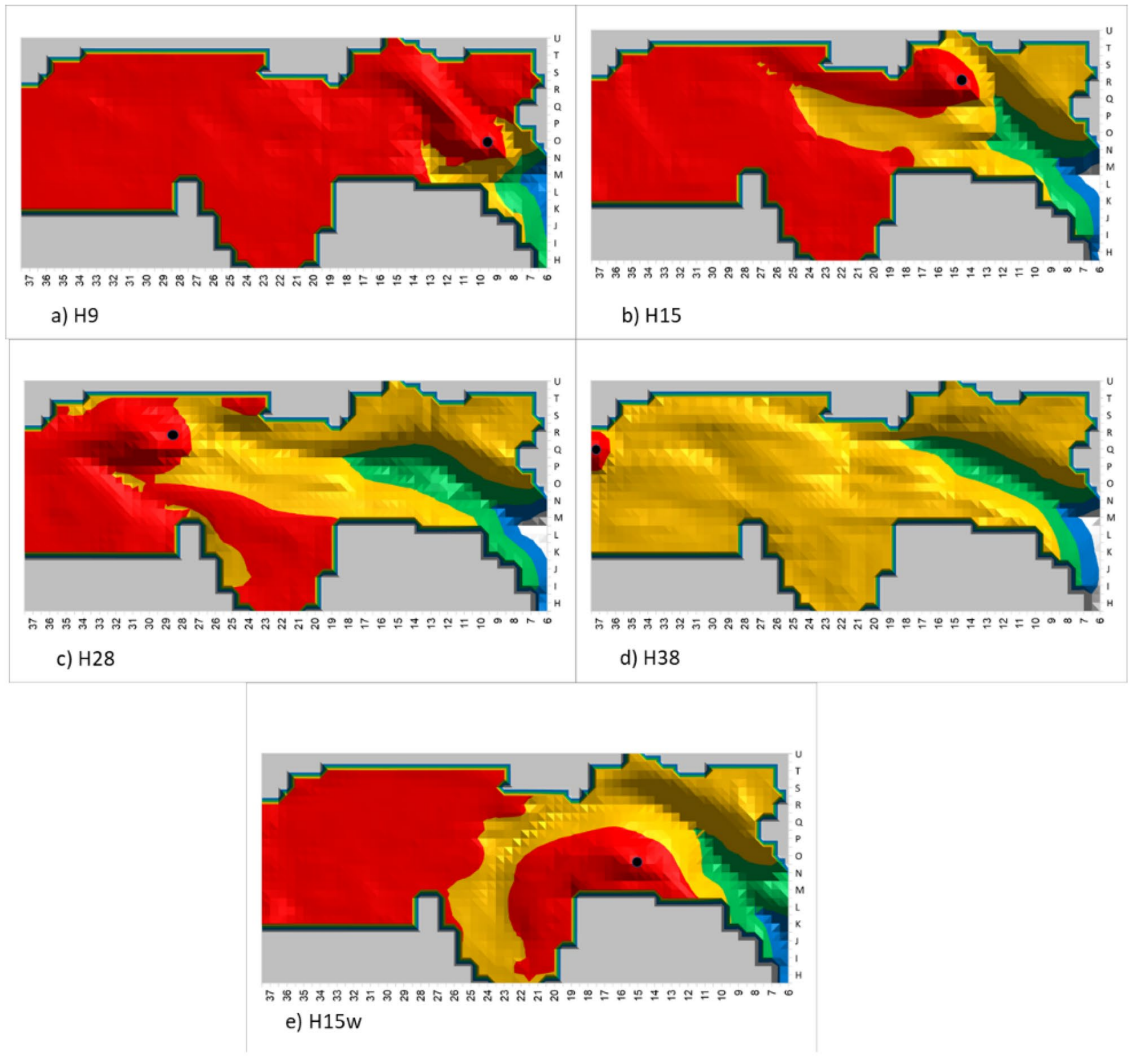
Five simulated hearth locations (black circle), with smoke density sensors at a height of 1 m. The green and blue zones are the long-duration occupation areas. The red zone is the non-occupational area. The yellow zone is the short-duration occupation area.

Simulation results for the three sensor heights (1.5 m, 1 m and 0.5 m) are presented, correspondingly, in Table 1 (standing height), Table 2 (sitting height) and Table 3 (sleeping height). The presented area size in the long-duration, short-duration, and non-occupational areas is the sum of the 0.5 × 0.5m^2^ squares. The long-duration occupation area distance from the hearth is the calculated distance in meters from the hearth to the closest green square. Figure 5 show examples of five simulation results for sensors placed at a height of 1 m.

**Table 1:** Results for smoke density from sensors at 1.5 m height.

Location	Long-duration occupation area size in 0.5 m^2^ (green and blue zones)	Short-duration occupation size in 0.5 m^2^ (yellow zone)	Non-occupational area size in 0.5 m^2^ (red zone)	Long-duration occupation area distance from hearth
H38	124	1020	17	21 m
H33	124	461	570	16 m
H33w	122	565	464	18 m
H28w	135	585	436	12 m
H28	153	401	600	11.5 m
H23w	122	610	421	8.5 m
H23	126	297	732	8.5 m
H20w	117	779	260	7 m
H20	128	314	713	5 m
H18w	127	517	531	5.5 m
H18	114	234	797	4.5 m
H17w	108	468	579	5 m
H15	100	243	813	3 m
H15w	102	346	715	4.5 m
H12	63	266	806	1.5 m
H9	59	71	1033	1 m

**Table 2:** Results for smoke density from sensors at 1 m height.

Location	Long-duration occupation area size in 0.5 m^2^ (green and blue zones)	Short-duration occupation size in 0.5 m^2^ (yellow zone)	Non-occupational area size in 0.5m^2^ (red zone)	Long-duration occupation area distance from hearth
H38	117	1014	12	19.5 m
H33	148	577	431	13 m
H33w	130	661	360	13 m
H28w	144	743	268	7.5 m
H28	155	495	500	9.5 m
H23w	136	701	318	5 m
H23	142	326	685	6 m
H20w	122	803	230	5.5 m
H20	126	328	701	3.5 m/3 m
H18w	128	570	454	3 m
H18	125	229	785	3.5 m
H17w	126	543	496	3.5 m
H15	108	296	750	2.5 m
H15w	102	384	677	3.5 m
H12	71	279	788	1.5 m
H9	58	55	1050	0.5 m

**Table 3:** Results for smoke density from sensors at 0.5 m height.

Location	Long-duration occupation area size in 0.5 m^2^ (green and blue zones)	Short-duration occupation size in 0.5 m^2^ (yellow zone)	Non-occupational area size in 0.5m^2^ (red zone)	Long-duration occupation area distance from hearth
H38	118	1003	14	20.5 m
H33	135	711	303	15.5 m
H33w	129	733	287	13.5 m
H28w	152	821	180	8.5 m
H28	146	594	410	10 m
H23w	148	753	250	6 m
H23	164	407	581	5 m
H20w	124	815	210	4.5 m
H20	124	354	664	3 m
H18w	129	570	441	1 m
H18	128	258	751	3 m
H17w	110	625	418	3 m
H15	107	294	751	2.5 m
H15w	98	435	625	3 m
H12	74	302	761	1 m
H9	59	63	1040	1 m

The results show that for a simulated hearth located from H23 up to the cave back wall at H38, the approximate long-duration occupation area for standing height (1.5 m) can be calculated as the sum of 122–153 squares, each with an area of 0.5 × 0.5 m^2^. For sitting height (1 m), this area is the sum of 140–150 such squares, and for resting/sleeping height (0.5 m), it is the sum 130–164 such squares. The long-duration occupation area distance from the hearth is between 8.5 and 21 m for standing height, 5–19.5 m for sitting height, and 6–20.5 m for resting height. For simulated hearth location in squares H15 to H20, the long-duration occupation area size for standing height can be calculated as the sum of 100–128 squares, each with an area of 0.5 × 0.5 m^2^. For sitting height, this area is the sum of 100–120 such squares, and for resting height it is the sum of 100–124 such squares. The distance of this area from the hearth is about 5 m for standing height, 3.5 m for sitting height, and 3 m for resting height. For simulated hearth location from the cave entrance up to H15 (not included), the long-duration occupation area size is less than 75 squares, with a distance of about one meter from the hearth for all three heights.

## Discussion

This paper presents our attempt to correlate between the results of smoke density simulations and archaeological activity areas as reconstructed at Lazaret cave by the excavators^72^. We begin our discussion section by considering hearth location in relation to the potential long-duration occupation area and its distance from the hearth. Afterwards, we describe the correlation between the simulated smoke density zones and the archaeological activity areas as reconstructed for archaeostratigraphic unit UA25. Finally, we briefly analyze the location of hearths at other archaeostratigraphic units of Lazaret cave in order to provide a broader perspective on hearth placements in the cave.

**Figure 6:**
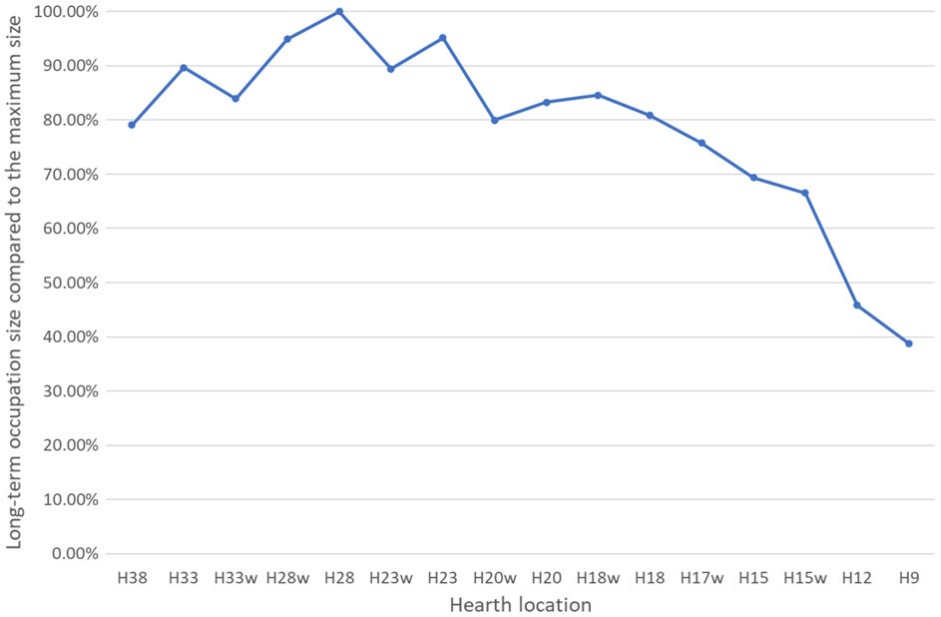
Comparison of the average long-duration occupation area for the three sensor heights, normalized to maximum area size.

We assume that the hearth in UA25 was activated for long durations, following the excavators' interpretation that the hearth was used for smoking meat^64^. Fire activity duration in hunter-gatherer groups around the world as described by ethnographers shows that 23 out of 28 groups kept their cooking fire burning continuously while 5 groups extinguished the fire at night. In addition, they found that 23 out of 33 groups used the hearth for warmth throughout the year without distinguishing the fire^46,95^. We compared average size of the area available for long-duration occupation (green and blue zones) for standing, sitting, and resting activities, as simulated by the three sensor heights, for all the simulated hearth locations, as shown in Fig. 6. It seems that the largest potential long-occupation area is available when the hearth is at the back of cave (H23 to H38). The second largest area is available when a hearth is located in squares H15-H20, and the smallest long-duration occupation area is available when the hearth is near the cave entrance, as presented in Tables 1, 2, and 3. The excavators located the hearth in UA25 at H15 (middle cave area), which means that the long-duration occupation area size was probably not the only parameter influencing the placement decision.

Early human activities such as cooking, smoking meat, keeping warm, and utilizing the available light require proximity to the hearth, and thus we investigated hearth distance from the potential long-duration occupation areas. Results indicate that for all sensor heights the largest distance between the hearth and the long-duration occupation area is for the hearth locations in squares H23 to H38, the middle range distance is for hearth locations in squares H15 to H20, and the shortest-range distance is for hearth locations up to H15, the actual location of the archaeological hearth.

**Figure 7:**
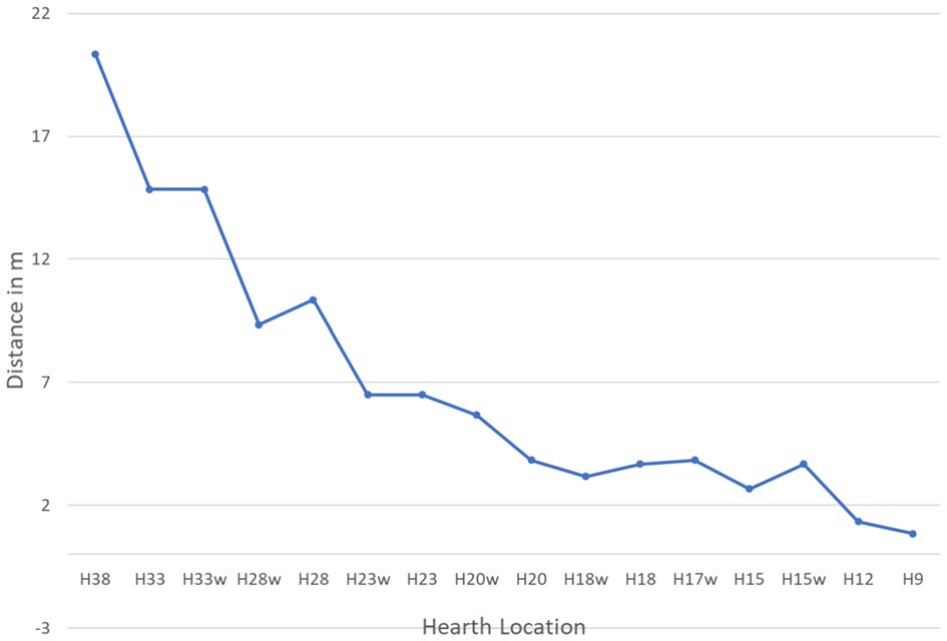
Average distance between hearth location and long-duration occupation area.

Experiments have shown that in an open-air site, warmth from the hearth begins to decrease at a distance of ca. 3 m^84^, and in a cave at a distance of ca. 6 m^58^. Thus, after about 6 m, the surrounding temperature is no longer influenced by the hearth. For all positionings (standing, sitting, and sleeping), when the hearth is located at H9-H20, there is less than 6 m between it and the long- duration occupation area (Fig. 7). For hearths located in the back-cave area (H23-H38), the distance to the long-duration occupation area is 6.5–20.3 m, and thus the hearth does not heat this area (Fig. 7). For the middle cave area (H15-H23), the distance to the long-duration occupation area is 3.6–5.6 m. For the cave entrance, this distance is 0.8–1.3 m (up to H15). Thus, a hearth located in either of these locations will heat the long-duration occupation area.

We suggest that hearth location should be chosen according to the potential long-duration occupation area along with its distance from the hearth in order to benefit from the hearth's advantages and functionalities while decreasing smoke exposure. When considering the largest long-duration occupation area, the best hearth locations are at the back of the cave (H23-H38). However, when considering hearth proximity, the best locations are at H9-H20, from the cave entrance up to the middle.

zone. When the two criteria are combined, however, we can see that the best hearth location is at one of the following: H20, H18w, H18, H17w, H15 and H15w for all three activity heights simulated by our sensors.

**Figure 8:**
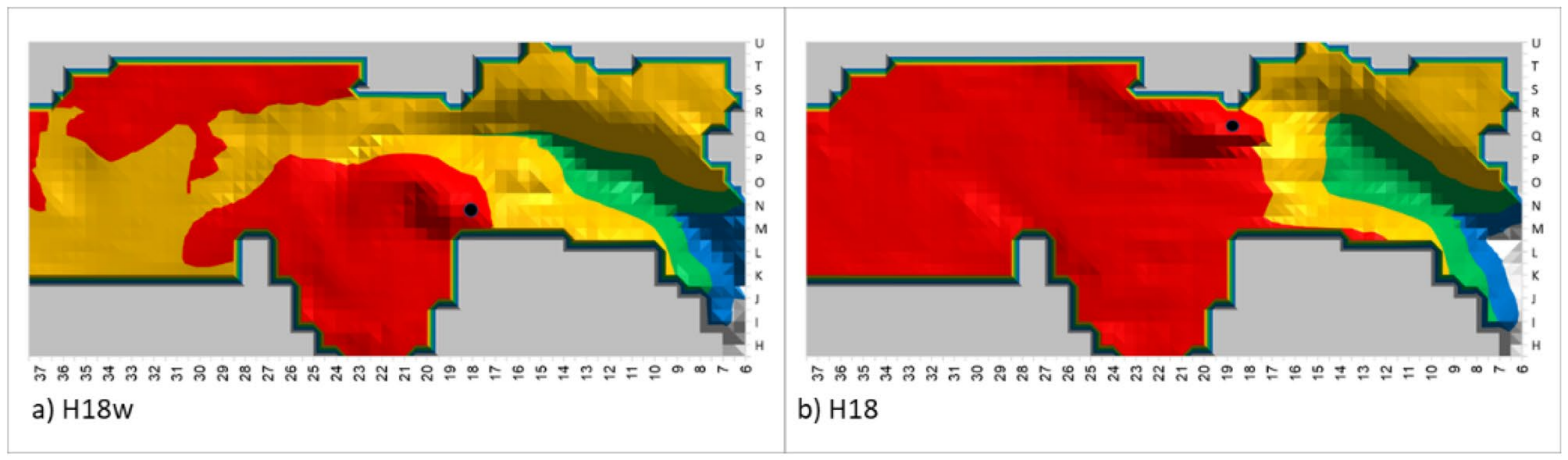
Simulation of smoke density for hearth locations H18w & H18 (black circle) with sensor height of 1 m. The green and blue zones are the long-duration occupation areas. The red zone is the non-occupational area. The yellow zone is the short-duration occupation area.

Another important parameter in planning human activity in a cave is ease of movement and interaction in the long-duration occupation area. For example, in Fig. 8a, where the hearth is located at H18w, the distance from the hearth to the long-duration occupation area is about 3 m, but at the same time the green zone area is very narrow and the area between the green zone and the hearth is dark yellow, indicating high smoke density. In comparison, a hearth located at H18, shown in Fig. 8b, provides a wider green zone along with a more limited yellow zone between the green zone and the hearth. A hearth at this location would thus provide the inhabitants with a larger green space in which to enjoy the hearth's warmth along with low smoke density. Table 4 presents the average number of 0.5 × 0.5m^2^ long-duration occupation squares that are located up to 5 m from the hearth. We can see that the long-duration occupation area providing the largest area of warmth is for hearth locations at H18, H15, H12 and H9. Thus, for the best results, the structure of the long-term occupation zone should be considered along with its size and distance from the hearth.

**Table 4:** Average long-duration occupation size with warmth effect.

Location	Avg number of 0.5 m^2^ long-duration occupation in squares up to 5 m from the hearth
H38, H33, H33w, H28, H23w, H23, H20w	0
H20	4
H18w	7
H18	10
H17w	5
H15	12
H15w	8
H12	30
H9	42

Once these three criteria are considered in tandem, only two optimal hearth locations remain: H18 and H15. Therefore, in UA25 at Lazaret Cave, the optimal hearth location is limited to an area of less than 5 × 5 m^2^. The actual hearth location is at H15, demonstrating that the Acheulian inhabitants of the cave chose this location carefully, in order to best take advantage of the benefits of the hearth.

Next, we try to correlate the proposed archaeological activity areas^72^ of UA 25 with the simulated smoke density of a hearth located at H15. The activity areas of UA25 are shown in Fig. 2. In bone breakage areas A and B, the inhabitants performed tasks that probably required extended occupancy of several hours^72^. This is also the case for areas I1 and I2, the proposed resting/sleeping areas^64^. Our simulation results indeed show that areas A, B, I1 & I2 are located in the long-duration occupation zone (blue & green zones). In those areas, early humans could stay comfortable and healthy for long periods of time while the hearth was active.

Along the east wall of the cave, areas H, C and Ct, characterized by accumulated food waste^72^, were probably waste disposal areas, as was the Et heap, with its large untreated bones and culinary remains^64^. Thus, these areas were probably used for short durations, as corroborated by the simulated smoke density. Early humans could remain in these areas for a limited time to execute specific tasks.

In Area E, another butchery and bone breaking locality, smoke density is just above the WHO and EPA recommendations, meaning that it is habitable for medium durations.

Area G, the meat smoking and drying area located behind the hearth, is a red zone, which correlates nicely with the suggested activity. In this case we can speculate that early humans might have smoked fresh meat in this location prior to fire ignition, and then returned to collect the smoked meat after the hearth was no longer operating.

**Figure 9:**
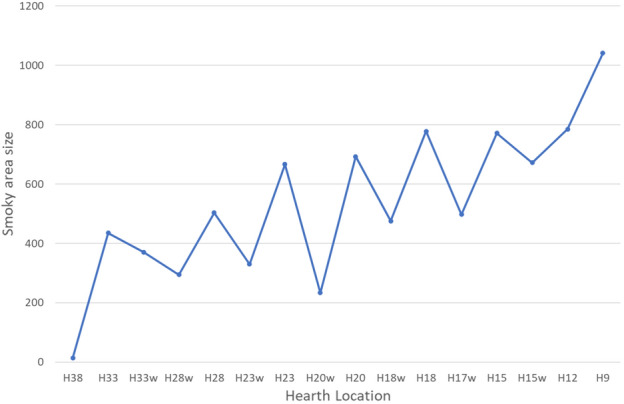
Comparison of red zone size for the various hearth locations.

A comparison of red zone size for the different simulated hearth locations shows that the red zone is larger when the hearth is located at the back of the cave than at the entrance, as shown in Fig. 9. The figure also shows that hearth placement on the east side of the cave (H15, H18, H20, H23, H28, H33) results in a larger red zone than on the west side (H17w, H18w, H20w, H23w, H28w). Figure 5 presents an example of this phenomenon: the simulated hearth located in square H15w is indeed smaller than the one located in square H15. Thus, placing the hearth in H15 results in one of the largest red zones, creating an area suitable for activities such as meat smoking, for which high smoke density is required.

Overall, the simulations of UA25 reveal an optimal hearth location zone of about 5 × 5 m^2^, which results in a balanced tradeoff between the long-duration occupation size, distance from hearth, and smoking area size around H15–H18, where the actual archaeological hearth was indeed found.

**Figure 10:**
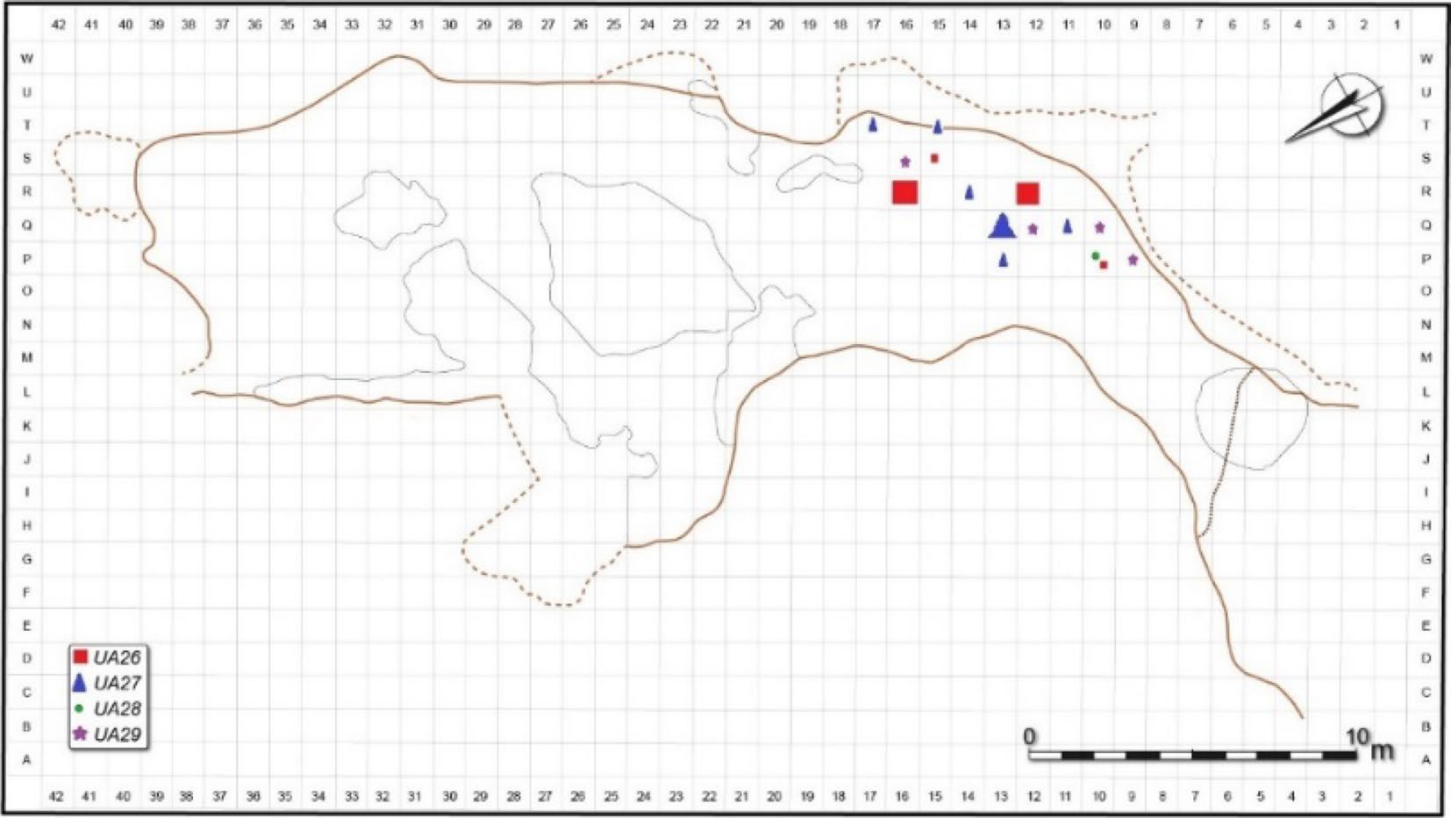
Hearth location in Lazaret Cave from various archaeostratigraphic units, based on^65^. (Courtesy of Prof. Henry de Lumley).

**Figure 11:**
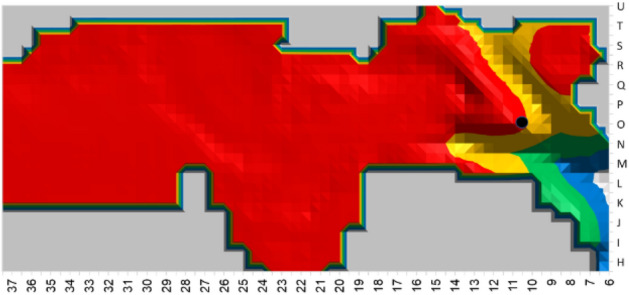
Simulation results for a small hearth at hearth location at H9 (black circle) with sensors at height 1.5 m. The green and blue zones are the long-duration occupation areas. The red zone is the non-occupational area. The yellow zone is short-duration occupation area.

To complete the picture, we analyze hearth locations found in other archaeostratigraphic units in Lazaret cave, as shown in Fig. 10. Three of the four small hearths in UA26^65^ were located in the 5 × 5 m^2^ area shown by our simulations to be the preferred zone. Although the hearth in square P10 (simulated as H9 hearth in Fig. 11), the long-duration occupation size is smaller than for the preferred location, the area can still be used for warmth since its entire long-duration occupation area distance from the hearth is less than 5 m.

Most of the hearths found in UA27^65^ are also located in the preferred zone. The hearth near the entrance (square Q11) is a small one, similar to the hearth in UA26. The single hearth in UA28^65^ was located similarly to the small hearths at UA26 and UA27. In UA29^65^ as well, the hearth locations are similar to those of UA26. It can be seen that the hearths were usually located in the preferred zone of H13-H17, but a smaller hearth found closer to the cave entrance near the simulated hearth at H9 still enabled warmth in the entire long-duration occupation area, albeit with a smaller occupation area size. Thus, it seems that our suggested hearth location parameters are appropriate for all occupation levels at Lazaret Cave. According to the excavators of the site, it is possible that multiple hearths were maintained simultaneously, as in archaeostratigraphic unit UA 29 (de Lumley, personal communication).

## Conclusion

Our simulations of smoke density at Lazaret Cave clearly show that Lower Paleolithic humans in this cave were able to choose the perfect locations for their hearths. This ability is the reflection of experience, ingenuity, and planned actions. The acquaintance of early humans with the effects of operating a hearth inside Lazaret cave is nothing short of astonishing, as are the clever and thoughtful considerations that went into organizing the space inside the cave. Our smoke simulations confirm the accuracy of the excavators' reconstruction, as the suggested activity areas correlate well with the smoke dispersal parameters. The possibility that several hearths were operated in parallel also needs to be explored. The effect of parallel hearths on smoke density and activity areas will be dealt with in a future paper, in which we will use our methodology developed here to simulate these more complex scenarios.
